# Formation of Asymmetric and Symmetric Hybrid Membranes of Lipids and Triblock Copolymers

**DOI:** 10.3390/polym12030639

**Published:** 2020-03-11

**Authors:** Hsiang-Chi Tsai, Yan-Ling Yang, Yu-Jane Sheng, Heng-Kwong Tsao

**Affiliations:** 1 Department of Chemical Engineering, National Taiwan University, Taipei 106, Taiwan; 2 Department of Chemical and Materials Engineering, National Central University, Jhongli 320, Taiwan

**Keywords:** lipid/triblock hybrid membrane, bridge- and loop-shape, symmetric or asymmetric membrane, dissipative particle dynamics

## Abstract

Hybrid membranes formed by co-assembly of A_x_B_y_A_x_ (hydrophilic-hydrophobic-hydrophilic) triblock copolymers into lipid bilayers are investigated by dissipative particle dynamics. Homogeneous hybrid membranes are developed as lipids and polymers are fully compatible. The polymer conformations can be simply classified into bridge- and loop-structures in the membranes. It is interesting to find that the long-time fraction of loop-conformation ($f_{L}$) of copolymers in the membrane depends significantly on the hydrophilic block length (x). As x is small, an equilibrium $f_{L}^{*}$ always results irrespective of the initial conformation distribution and its value depends on the hydrophobic block length (y). For large x, $f_{L}$ tends to be time-invariant because polymers are kinetically trapped in their initial structures. Our findings reveal that only symmetric hybrid membranes are formed for small x, while membranes with stable asymmetric leaflets can be constructed with large x. The effects of block lengths on the polymer conformations, such as transverse and lateral spans ($d^{⊥}$ and $d^{\|}$) of bridge- and loop-conformations, are discussed as well.

## 1. Introduction

Amphiphilic molecules such as lipid and block copolymers possess both hydrophilic and hydrophobic moieties. In selective solvents, they can self-assemble into various ordered structures, including worm-like or spherical micelles [1], membranes [2,3], and vesicles [4,5]. Vesicles composed of lipids are called liposomes, while those constituted of block copolymers are referred to as polymersomes. Liposomes have been extensively studied and applied to the field of biomedicine, such as drug delivery [6,7,8]. Although liposomes have good biocompatibility, low toxicity, and superior encapsulation efficiency of active elements, their low mechanical stability limits the applications [9,10]. In contrast, polymersomes have good mechanical stability and chemical versatility [4,11], and can be composed of diblock copolymers [4,12,13], triblock copolymers [14,15,16], or dendrimers [17,18]. However, their applicability is often hindered by the low permeability of the polymeric membrane [19]. It is known that co-assembly of block copolymers with typical lipids can counteract the weakness of liposomes and polymersomes by preserving the lipid membrane properties and at the same time strengthening the membrane [20,21,22,23,24,25,26,27,28]. In other words, hybrid membranes have the advantage of possessing both the biocompatibility of lipids and the mechanical stability of block copolymers.

Membranes formed by pure diblock copolymers are bilayered [29] while monolayered, bilayered, or mixed membranes can be developed by triblock copolymers [30,31]. It is known that A_x_B_y_A_x_ triblock copolymers can exhibit two possible conformations, U- (loop) or I- (bridge) shape, in the polymer membrane [32,33,34,35,36]. The triblock copolymer is termed the loop-conformation as its two hydrophilic moieties are on the same side of the membrane, while a copolymer is called a bridge-shape copolymer when its two hydrophilic blocks situate on the opposite sides of the membrane. It was revealed that the conformational entropy of loop-shape is greater than that of bridge-shape, and thus the equilibrium fraction of the former is higher [30]. The equilibrium fraction is independent of the initial composition and insensitive to the immiscibility between hydrophilic and hydrophobic blocks (χ-parameter). However, the relaxation time grows very rapidly with χ, indicating that the kinetically trapped fraction of a conformation can be controlled by strong A-B immiscibility [30,31].

Most of the lipid/polymer hybrid membranes were formed symmetrically [22,37,38,39,40] and both leaflets of the membrane are alike. In the recent work of Kang et al. [40], it was shown that lateral co-assembly of a small amount of amphiphilic triblock copolymers poly(ethylene oxide)-b-poly(ε-caprolactone)-b-poly(ethylene oxide) can indeed significantly improve the stretching modulus of giant unilamellar vesicles of 1,2-dipalmitoyl-sn-glycero-3-phosphocholine. Their work also indicated that the triblock copolymers tend to form a loop-shape rather than a bridge-shape conformation [40]. In contrast to symmetric membranes, the biological membranes frequently exhibit asymmetric distribution of constituents on two leaflets, which plays an essential role in the membrane properties and functions [41]. In fact, the uneven distribution of two types of lipids in the two leaflets affects the membrane properties such as curvature, stability, and permeability [41,42]. Consequently, the asymmetric distribution of polymers on two leaflets in the hybrid membrane is also expected to have an impact on the membrane properties. Recently, the formation of an asymmetric giant hybrid unilamellar vesicle which has a lipid monolayer on the outside and a polymer monolayer on the inside has been demonstrated [41]. Its stability with time was followed by monitoring transverse diffusion (flip-flop) of lipids. Evidently, the construction of an asymmetric membrane and the increase of its stability are of great importance and represent key challenges [41].

Since the A_x_B_y_A_x_ triblock copolymers can exhibit two possible conformations, loop or bridge, the formations of asymmetric lipid/triblock hybrid membranes are inherently more complicated than their lipid/diblock counterparts [29,30,31]. The lipid/triblock hybrid membrane is called symmetric as the fractions of the loop-shape copolymers in the upper and lower leaflets are the same, and is designated asymmetric otherwise. Note that the bridge-shape triblocks always exist at both leaflets simultaneously. In this work, a dissipative particle dynamics (DPD) method was employed to study both symmetric and asymmetric hybrid membranes. They can be easily created initially by evenly or unevenly distributing triblock copolymers into the two leaflets of lipid membranes. In general, asymmetric membranes are thermodynamically unstable due to the entropic effect. Thereby, the flip-flops of polymers between two leaflets and shape transformations between loops and bridges were monitored and the steady-state/equilibrium compositions of the two leaflets were determined. The effect of hydrophilic block length (x) on the stability of the asymmetric membranes was investigated and the prerequisite to acquire stable asymmetric hybrid membrane was proposed. Finally, the dependence of the equilibrium fraction of loop conformation on the hydrophobic block length (y) was studied as well.

## 2. Model and Simulation Method

**Figure 1:**
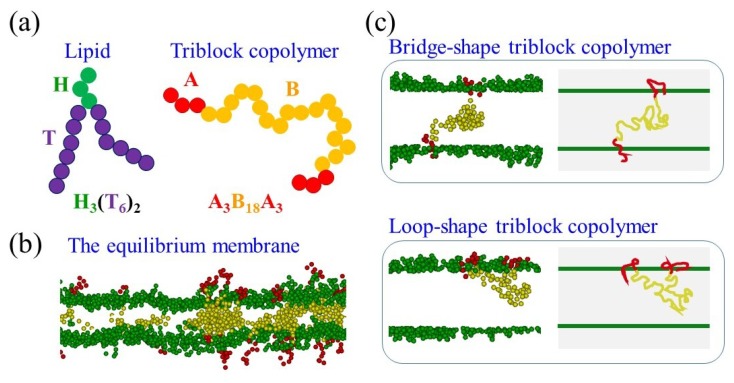
(**a**) Schematics of model lipid and triblock copolymer, (**b**) the sliced snapshot of the equilibrium hybrid membrane, and (**c**) snapshots and schematics of the bridge-shape and the loop-shape triblock copolymers. Note that in (**b**) and (**c**), lipid tails are omitted. Green and purple beads represent lipid head and tail, respectively. Red and yellow beads denote hydrophilic and hydrophobic parts of the copolymer, respectively.

DPD is a mesoscale simulation and obeys Newton’s equation of motion [43,44,45,46,47]. Each DPD bead encloses an assembly of atoms or molecules into a distinct coarse-grained unit. Three main forces acting on a DPD bead are conservative, dissipative, and random forces, all of which are pairwise-additive and short-ranged [48]. The conservative force $F^{C}$ is soft-repulsive and the cutoff radius is $r_{c}=1$ beyond which it ceases to exist,

(1) $$F_{ij}^{C}=\{\begin{array}{cc} a_{ij}\left(r_{c}-r_{ij}\right){\hat{r}}_{ij}, & r_{ij}<r_{c}\\ 0, & r_{ij}>r_{c} \end{array},$$

where $r_{ij}$ denotes the magnitude of the vector (${\vec{r}}_{ij}$) between beads *i* and *j* and ${\hat{r}}_{ij}={\vec{r}}_{ij}/r_{ij}$ represents the unit vector. The parameter $a_{ij}$ stands for the maximum repulsion strength between beads *i* and *j*. For the identical type of beads, $a_{ii}=25$ is chosen to yield the compressibility of the DPD fluid the same as water [48]. $a_{ij}$ increases from 25 as beads *i* and *j* become more incompatible. The detailed descriptions of dissipative and random forces have been given elsewhere [43,48]. The model lipid and triblock copolymer used in this work are shown in Figure 1a. A spring force $F_{ij}^{S}=-\underset{j}{\sum }k_{s}\left(r_{ij}-r_{eq}\right){\hat{r}}_{ij}$ exists between the bonded DPD beads, where $k_{s}=100$ and the equilibrium length is set as $r_{eq}=0.4$ for lipids and $r_{eq}=0.7$ for copolymers [30,49]. Moreover, the bending force with the bending constant $k_{\theta }=5$ is imposed to the lipid tails to fortify their rigidity. Our model lipid resembles the structure of a palmitoyl-oleyl-sn-phosphatidylcholine which consists of one saturated (straight) tail and one with unsaturated (bent) tail. The combination of the saturated and unsaturated features adds to the fluidity of the tails that are constantly in motion. For saturated tails, the angle between two consecutive bonds is constrained to be close to the value of π. For unsaturated tails, another bending force is imposed to imitate the kink structure with an equilibrium bond angle of 2π/3 [29].

An equilibrium planar membrane as shown in Figure 1b was acquired by the NPγT ensemble with fixed number of beads, constant pressure, surface tension, and temperature [50]. The Langevin piston approach [51] was employed to keep the membrane at zero tension by varying the width and depth of the simulation box (L_x_ and L_y_). The height of the box (L_z_) changed accordingly to maintain the density of the system. The hybrid membranes were formed by H_3_(T_6_)_2_ lipid and A_x_B_y_A_x_ triblock copolymer immersed in the selective solvent (S). As shown in Figure 1a, H and A represent hydrophilic beads, while T and B depict hydrophobic beads of lipids and copolymers, respectively. Note that the molecular weight of the polymer is proportional to the chain length or bead number (i.e., (2x + y) for our triblock copolymer (A_x_B_y_A_x_)). The self-interaction parameters are always set as $a_{ii}=25$ for *i* = H, T, A, B, and S. A typical lipid bilayer can be developed by setting $a_{HT}=50$. In this work, the copolymer has weakly incompatible A- and B-blocks, therefore one assumes $a_{AB}=35$. To ensure a homogeneous hybrid membrane, the lipids and copolymers are set to be fully compatible (i.e., H and A are similar beads and T and B are alike as well). As a result, one chooses $a_{HA}=a_{BT}=25$ and $a_{HB}=a_{AT}=35$ and their interactions with the selective solvent are also the same as $a_{HS}=a_{AS}=25$ and $a_{TS}=a_{BS}=50$. Note that the same DPD approach can be used to study polymers or lipids of any kind. However, the interaction parameters ($a_{ij}$) and molecular lengths in the DPD approach should vary with the types of polymers or lipids in the studied systems. The initial configurations of the hybrid lipid/copolymer membrane are constructed by forming a lipid membrane first and then incorporating triblock copolymers into the membrane with different conformations. The conformations of the triblock copolymers can be classified into two types: bridge- and loop-shapes as demonstrated in Figure 1c. For a copolymer with its two A-blocks situating on the opposite sides of the membrane, it is called a bridge-shape copolymer. On the other hand, a loop-shape copolymer is identified for both A-blocks positioning on the same side of the membrane. For sufficiently short triblock copolymers, the bridge conformation looks like “I” shape, while the loop conformation resembles “U” shape.

Our simulation system contained approximately 150,000 DPD beads in a box with the initial size about 37 × 37 × 37. Periodic boundary conditions were employed in all three spatial directions. The velocity Verlet scheme was adopted to integrate the equation of motion and the time increment was chosen as Δt=0.01. Equilibrium tenionlesss membranes were acquired after 2 × 10^6^ to 2 × 10^7^ time-steps. All the units were nondimensionalized by the cutoff distance r_c_, bead mass m, and temperature k_B_T, and all of which were set to unity.

The volume fractions of water and the hybrid membrane were about 84% and 16% of the total system, respectively, in our simulation system. Within the hybrid membrane, the volume fractions of copolymers ($\varphi_{p}$) and lipids ($\varphi_{l}$) were defined as

(2) $$\varphi_{p}=\frac{\mathrm{total}\;\mathrm{number}\;\mathrm{of}\;\mathrm{polymer}\;\mathrm{beads}\;\mathrm{in}\;\mathrm{the}\;\mathrm{membrane}}{\mathrm{total}\;\mathrm{number}\;\mathrm{of}\;\mathrm{beads}\;\mathrm{in}\;\mathrm{the}\;\mathrm{membrane}},$$

(3) $$\varphi_{l}=\frac{\mathrm{total}\;\mathrm{number}\;\mathrm{of}\;\mathrm{lipid}\;\mathrm{beads}\;\mathrm{in}\;\mathrm{the}\;\mathrm{membrane}}{\mathrm{total}\;\mathrm{number}\;\mathrm{of}\;\mathrm{beads}\;\mathrm{in}\;\mathrm{the}\;\mathrm{membrane}}.$$

Note that $\varphi_{p}+\varphi_{l}=1$. As mentioned, a hybrid membrane was constructed by forming the lipid bilayer first and then incorporating the triblock copolymers with bridge-shape, loop-shape, or both conformations into the membrane. The numbers of loop- and bridge-shape copolymers in the membrane can be determined directly in simulations and are represented by $N_{L}$ and $N_{B}$, respectively. The total number of copolymers is $N_{t}=N_{L}+N_{B}$. Therefore, the fractions of loop and bridge conformations are defined as $f_{L}=N_{L}/N_{t}$ and $f_{B}=N_{B}/N_{t}$, correspondingly. In order to study the asymmetric membrane, the loop-shape copolymers in the upper and lower leaflets can be separately identified as $N_{L}^{up}$ and $N_{L}^{lo}$, respectively. Since $N_{L}=N_{L}^{up}+N_{L}^{lo}$, one has $f_{L}=f_{L}^{up}+f_{L}^{lo}$, where $f_{L}^{up}=N_{L}^{up}/N_{t}$ and $f_{L}^{lo}=N_{L}^{lo}/N_{t}$. Note that the hybrid membrane is termed symmetric as $f_{L}^{up}=f_{L}^{lo}$, and it is designated asymmetric as $f_{L}^{up}\neq f_{L}^{lo}$. Because $f_{B}^{up}$ always equal to $f_{B}^{lo}$, $f_{B}$ is irrelevant in the determination of the membrane asymmetry. Therefore, the evolutions of $f_{L}^{up}$ and $f_{L}^{lo}$ were monitored to examine the stability of an asymmetric membrane.

## 3. Results and Discussion

In this work, the hybrid membrane was formed by the co-assembly of H_3_(T_6_)_2_ lipids and A_x_B_y_A_x_ triblock copolymers. The influences of the lengths of the hydrophilic A-blocks on the steady-state/equilibrium structures of hybrid membranes were explored. The stability of asymmetric membranes was examined and the evolution toward symmetric membranes was monitored based on the fractions of the loop-shape copolymers in the upper and lower leaflets.

### 3.1. Formation of Stable Asymmetric Membranes by Long Hydrophilic A-Block

**Figure 2:**
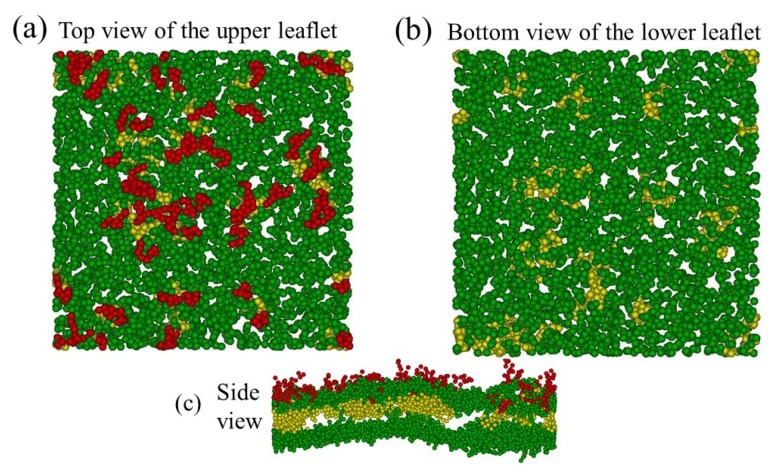
Representative snapshots of the equilibrium asymmetric membrane. (**a**) Top view of the upper leaflet. (**b**) Bottom view of the lower leaflet. (**c**) Side view of the membrane. Note that only loop-shape copolymers are shown and all bridge-shape copolymers and lipid tails are omitted. Green, red, and yellow beads represent lipid head, hydrophilic and hydrophobic beads of the copolymer, respectively.

The properties of the triblock copolymer-hybridized lipid membranes rely significantly on the triblock copolymer structures within the hybrid membrane. They are generally characterized by the loop and bridge conformations of copolymers. Here, A_10_B_56_A_10_ copolymers were employed to co-assemble with the lipid H_3_(T_6_)_2_ bilayers, and the volume fraction of copolymers was $\varphi_{p}=0.1$. Compared to the lipid tail (T_6_), the hydrophobic B-block of the copolymer (B_56_) is rather long. Moreover, the hydrophilic A-block (A_10_) of the copolymer is also regarded as a long block with respect to the lipid head (H_3_). An asymmetric hybrid membrane can be developed by setting up distinct initial configurations of the upper and lower leaflets, for example, $f_{L}^{up}=0.6$ and $f_{L}^{lo}=0$. That is, 60% of the total copolymers are loop-shape and only exist in the upper leaflet. Obviously, one has $f_{B}=1-f_{L}=0.4$ in this membrane where $f_{L}=f_{L}^{up}+f_{L}^{lo}$. Figure 2 shows representative snapshots of the steady-state asymmetric membrane in which bridge-shape copolymers are omitted. All loop-shape copolymers are illustrated in Figure 2a from the top view of the upper leaflet. Note that no phase separation is observed since lipids and copolymers were fully compatible in this work. In contrast, no loop-shape copolymers can be seen from the bottom view of the lower leaflet, as demonstrated in Figure 2b. Asymmetric characteristics of the membrane can also be clearly identified from the side view because the hydrophilic A-blocks (red beads) are only observed in the upper leaflet (Figure 2c).

**Figure 3:**
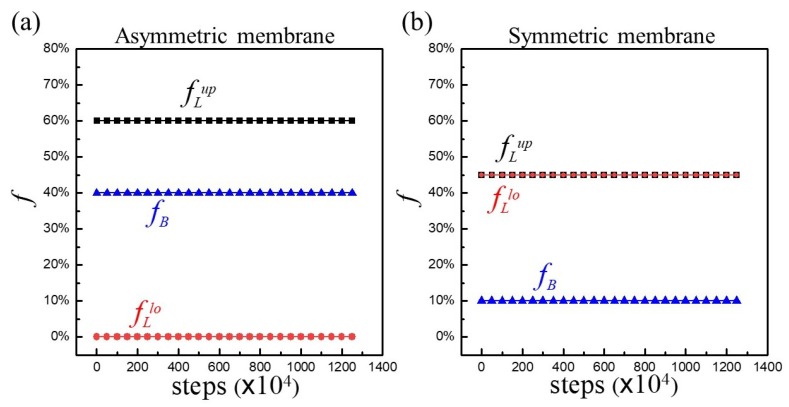
The evolutions of $f_{L}\;\left(f_{L}^{up};\;f_{L}^{lo}\right)$ and $f_{B}$ with respect to simulation steps for A_10_B_56_A_10_ in a hybrid membrane of $\varphi_{p}=0.1$ for (**a**) asymmetric and (**b**) symmetric membranes.

In general, asymmetric membranes are thermodynamically unstable due to entropic effect. However, the asymmetric characteristics of hybrid membranes with A_10_B_56_A_10_ persisted throughout the simulation process in the duration of 1.25 × 10^7^ time steps. Figure 3a illustrates the evolutions of $f_{L}^{up}$, $f_{L}^{lo}$, and $f_{B}$ with respect to simulation steps. As one can see, all f always remained the same as their initial values in this asymmetric membrane. For a symmetric membrane with $f_{L}^{up}=f_{L}^{lo}=0.45$ and $f_{B}=0.1$, the same time-invariant feature was also observed, as demonstrated in Figure 3b. The above consequences revealed that while copolymers were able to move around laterally, they failed to flip flop from one leaflet to another. Moreover, the conformational changes between loop- and bridge-shape were absent as well regardless of asymmetric or symmetric membranes.

According to the concept of entropy maximization, the swapping of copolymers between the two leaflets to attain a symmetric membrane is favorable through flip-flops. On the basis of the same underlying driving force, the interchange between loop and bridge conformations to achieve an equilibrium ratio ($f_{L}/f_{B}$) is anticipated as well. However, the aforementioned outcomes can be inhibited by energy barriers and an asymmetric hybrid membrane is formed. In the study of polymer membranes of triblocks (A_3_B_y_A_3_) which contain both loop and bridge conformations, the initial configurations can persist if the incompatibility between hydrophilic A-block and hydrophobic B-block is strong enough (e.g., $a_{AB}\geq 50$) [29,30]. That is, the hydrophobic layer of B-blocks provides a very strong resistance to the passage of hydrophilic A-blocks from one side to another side of the membrane. Note that the hydrophilic A-block is short (A_3_). However, in this work, the incompatibility between hydrophilic A-block and hydrophobic lipid tail ($a_{AT}=35$) was not strong. As a result, it is believed that the large A-block length is the cause of the invariance of the copolymer fractions in both leaflets over times. This finding signifies that by co-assembling triblock copolymers of long hydrophilic A-blocks into lipid bilayers, asymmetric membranes with distinct initial values of $f_{L}^{up}$ and $f_{L}^{lo}$ can be designed and exist stably for a long period of time.

**Figure 4:**
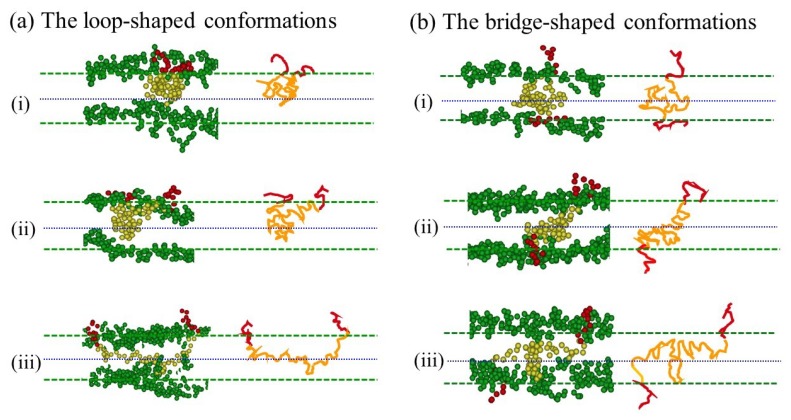
Snapshots and schematics of various (**a**) loop and (**b**) bridge conformations of the A_10_B_56_A_10_ in a hybrid membrane of $\varphi_{p}=0.1$. Green, red, and yellow beads represent lipid head, hydrophilic and hydrophobic beads of the copolymer, respectively.

Compared to the lipid molecule (H_3_(T_6_)_2_), the contour length of the triblock copolymer (A_10_B_56_A_10_) is rather long. As a result, a simple description of “U” or “I” shape is insufficient to describe various complicated conformations exhibited by triblock copolymers. Figure 4 illustrates various snapshots and schematics of the loop- and bridge-shape conformations taken from the asymmetric membrane of A_10_B_56_A_10_. It is evident that both loop- and bridge-shaped conformations can exist within the hybrid membrane and the conformations persist as time passes. Figure 4a clearly demonstrates that the hydrophobic B-block of loop-shape copolymers frequently passes through the midplane, and this scenario becomes more evident as the B-block length increases. In fact, approximately 30% of the B-block of each loop-shape copolymer was found to locate at the other leaflet of the lipid bilayer. For copolymers with much shorter B-block (A_10_B_18_A_10_), 16% of the B-block loop still resides across the midplane. Our outcomes do not agree with recent NMR findings of lipid/triblock hybrid membranes in which only loop-shape conformations were observed [40]. In their work, each copolymer does not penetrate across the midplane of the lipid bilayer and tends to stay beneath the lipid heads along the hydrophobic tail layer. This inconsistency may be attributed to the fact that in the experiment, the hydrophobic B-blocks are quite compatible with the lipid heads, leading to the absence of B-blocks in the central region of the lipid bilayer and the tendency of staying near the interface.

**Figure 5:**
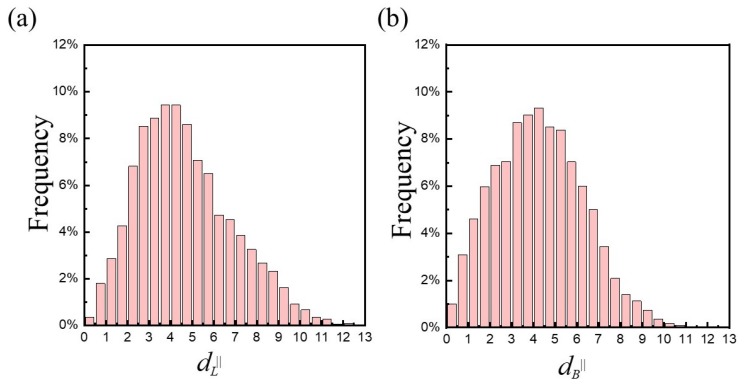
The distribution of the end-to-end distance parallel to the membrane surface for the (**a**) loop ($\langle d_{L}^{\|}\rangle$) and (**b**) bridge ($\langle d_{B}^{\|}\rangle$ ) conformations of the A_10_B_56_A_10_ in a hybrid membrane of $\varphi_{p}=0.1$.

The conformations of copolymers can be characterized by the end-to-end distances between the two end beads of the hydrophobic B-block in terms of $d^{\|}$ and $d^{⊥}$, corresponding to the distances parallel and perpendicular to the membrane interface, respectively. The mean values of the vertical end-to-end distance were about $\langle d_{L}^{⊥}\rangle =0.90\pm 0.20$ for loop-shape copolymers, and $\langle d_{B}^{⊥}\rangle \approx 4.71\pm 0.52$ for bridge-shape copolymers. The small value of $\langle d_{L}^{⊥}\rangle$ corresponded to the slightly undulated membrane. As expected, $\langle d_{B}^{⊥}\rangle$ was approximately equal to the thickness of the hydrophobic layer of the membrane. Unlike the vertical end-to-end distance, the horizontal counterpart exhibited a broader distribution, as shown in Figure 5. The distributions of $d_{L}^{\|}$ and $d_{B}^{\|}$ of long triblock copolymers were slightly right-skewed with a long tail, revealing diverse structures which cannot be simply described by “U” or “I” shape. The extent of expansion of triblock copolymers along the membrane plane is comparable for the loop- and bridge-shape conformations according to $\langle d_{L}^{\|}\rangle =4.67\pm 2.21$ and $\langle d_{B}^{\|}\rangle =4.27\pm 2.03$. On the basis of conformational entropy of a linear polymer, the propensity for the B-blocks to take a loosely coil-like structure surpasses their tendency to full extension (see Figure 4). 

### 3.2. Evolution toward Symmetric Membrane with Short Hydrophilic A-Block

**Figure 6:**
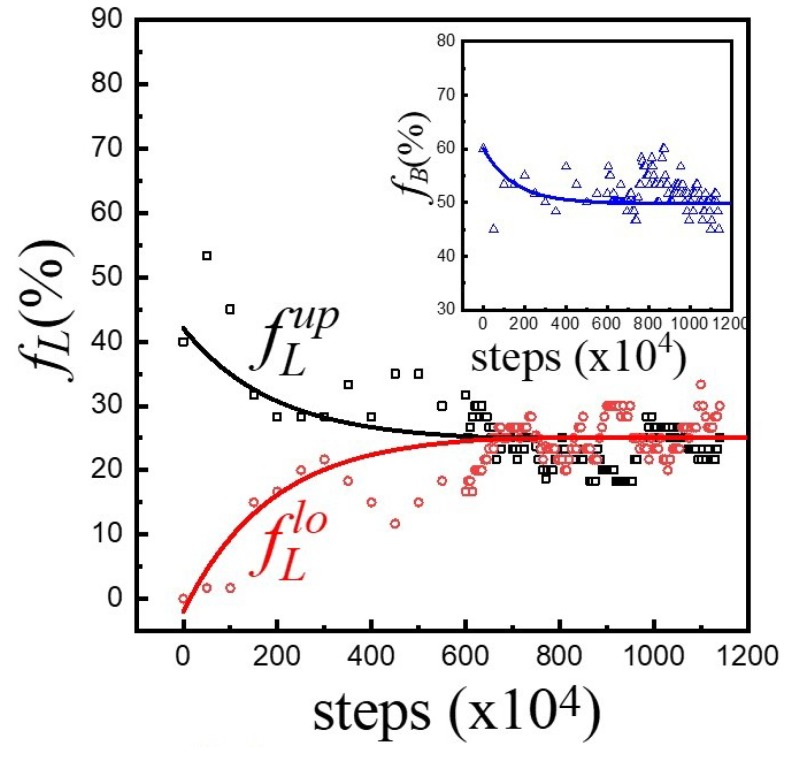
The evolutions of $f_{L}^{up}$, $f_{L}^{lo}$ and $f_{B}$ with respect to simulation steps for A_3_B_36_A_3_ in a hybrid membrane of $\varphi_{p}=0.1$.

We have shown that triblock copolymers with long hydrophilic A-blocks (A_10_B_56_A_10_) fail to flip flop and change conformation in the hybrid membrane, even though the incompatibility between A-blocks and lipid tails is weak ($a_{AT}=35$). One would wonder what happens if A-blocks become short. Therefore, hybrid membranes of lipids (H_3_(T_6_)_2_) and triblock copolymers (A_3_B_36_A_3_) were considered. First, an asymmetric membrane with $f_{L}^{up}\left(0\right)=0.4$, $f_{L}^{lo}\left(0\right)=0$, and $f_{B}\left(0\right)=0.6$ was initially constructed. Figure 6 shows the evolutions of $f_{L}^{up}\left(t\right)$ and $f_{L}^{lo}\left(t\right)$ with respect to simulation steps. While $f_{L}^{up}$ declined from 0.4, $f_{L}^{lo}$ grew from 0, unlike the invariant feature of A_10_B_56_A_10_ (cf. Figure 3). Eventually, they converged to the same value $f_{L}^{up,*}=f_{L}^{lo,*}=\frac{f_{L}^{*}}{2}=0.255$, indicating the formation of a symmetric membrane. That is, an asymmetric hybrid membrane of triblock copolymers with short hydrophilic A-blocks tends to evolve toward an equilibrium symmetric membrane. It is worth mentioning that $f_{B}\left(t\right)$ decreased from 0.6 to 0.49 as shown in the inset of Figure 6, while $f_{L}=f_{L}^{up}+f_{L}^{lo}$ increased from 0.4 to 0.51. This consequence implies that the evolution toward a symmetric membrane is accompanied with transformations between loops and bridges.

**Figure 7:**
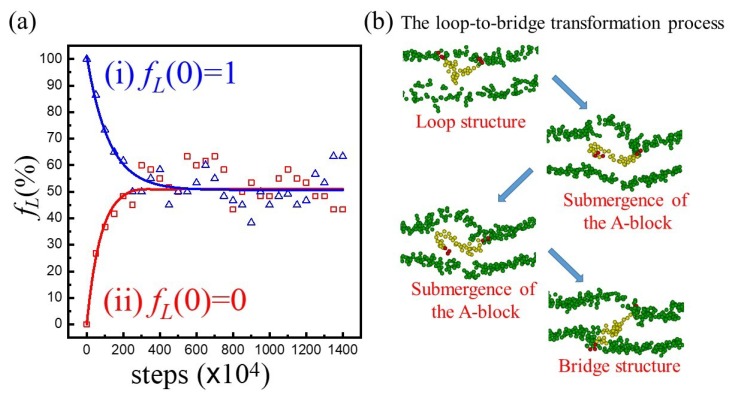
(**a**) The evolutions of $f_{L}$ with respect to simulation steps for A_3_B_36_A_3_ in a hybrid membrane of $\varphi_{p}=0.1$. (**b**) The illustration of the loop-to-bridge transformation process. Green, red, and yellow beads represent lipid head, hydrophilic and hydrophobic beads of the copolymer, respectively.

In order to distinguish evolution toward symmetry and conformation change, two types of symmetric hybrid membranes were initially constructed: (i) $f_{L}^{up}=f_{L}^{lo}=0.5$ (i.e., $f_{L}\left(0\right)=1$) and (ii) $f_{L}^{up}=f_{L}^{lo}=0$ (i.e., $f_{L}\left(0\right)=0$). Note that $f_{L}=0$ indicates that only bridge-shape copolymers exist in the hybrid membrane. Figure 7a illustrates the evolutions of $f_{L}$ with respect to simulation steps. As we can see, $f_{L}$ declined from $f_{L}\left(0\right)=1$ in case (i) and reached an equilibrium value ($f_{L}^{*}\approx 0.51$) eventually. Similarly, $f_{L}$ ascended from $f_{L}\left(0\right)=0$ in case (ii) and reached the same $f_{L}^{*}$ ultimately. These results clearly reveal that conformation changes of A_3_B_36_A_3_ do take place within the membrane. The representative loop-to-bridge transformation process is illustrated in Figure 7b. Initially, the copolymer took the loop-shape structure with both A-blocks on the surface of the upper leaflet. Then one of the A-block became submerged within the membrane. Finally, this A-block came out of the hydrophobic layer to the other side of the membrane and thus a bridge-shape chain was developed.

The above results show that the hybrid membranes with A_3_B_36_A_3_ always advance toward the symmetric configuration of $f_{L}^{*}\approx 0.51$ and $f_{B}^{*}\approx 0.49$, irrespective of the initial symmetric or asymmetric conditions. As a result, the final outcome is a true equilibrium state with symmetric configuration and specific $f_{L}^{*}$. This is due to the fact that the energy barrier of a short hydrophilic A-block moving across the hydrophobic layer is significantly smaller that of a long A-block. Thereby, it is much easier to observe flip-flops of loops from one leaflet to another and conformation interchange between loop- and bridge- structures. According to free energy minimization, a symmetric membrane can achieve maximum entropy in a fully compatible system and should be a favorable equilibrium outcome. The processes of flip-flops and conformation interchanges of the triblocks are driven by thermal fluctuations and have to overcome the energy barrier resulting from the contacts between hydrophilic A-blocks and hydrophobic lipid tails (T). The role of the length of hydrophilic A-blocks can be realized from the concept of the immiscibility of polymer blends [52]. The barrier is expected to be proportional to $x\chi_{AT}$, where x represents the hydrophilic A-block length. The χ parameter between A-blocks and lipid tails is proportional to the interaction parameter in DPD simulation, $\chi_{AT}∼a_{AT}$. Evidently, the energy barrier is expected to ascend as the incompatibility between A-blocks and lipid tails ($a_{AT})$ increases or A-block length (x) grows. Consequently, the hybrid membranes involving copolymers with long hydrophilic blocks (e.g., A_10_B_56_A_10_) tend to be kinetically trapped in their original configurations and conformations, and their final statuses are metastable states instead of true equilibrium states.

**Figure 8:**
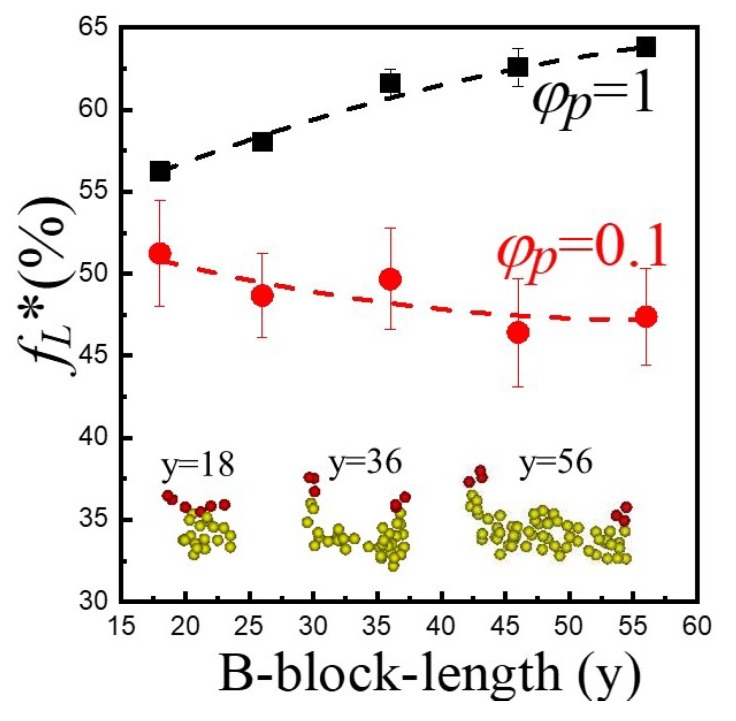
The variation of $f_{L}^{*}$ as a function of hydrophobic B-block length (y). The hybrid membrane is formed by lipids and A_3_B_y_A_3_ with $\varphi_{p}=0.1$. Red and yellow beads represent hydrophilic and hydrophobic beads of the copolymer, respectively.

**Figure 9:**
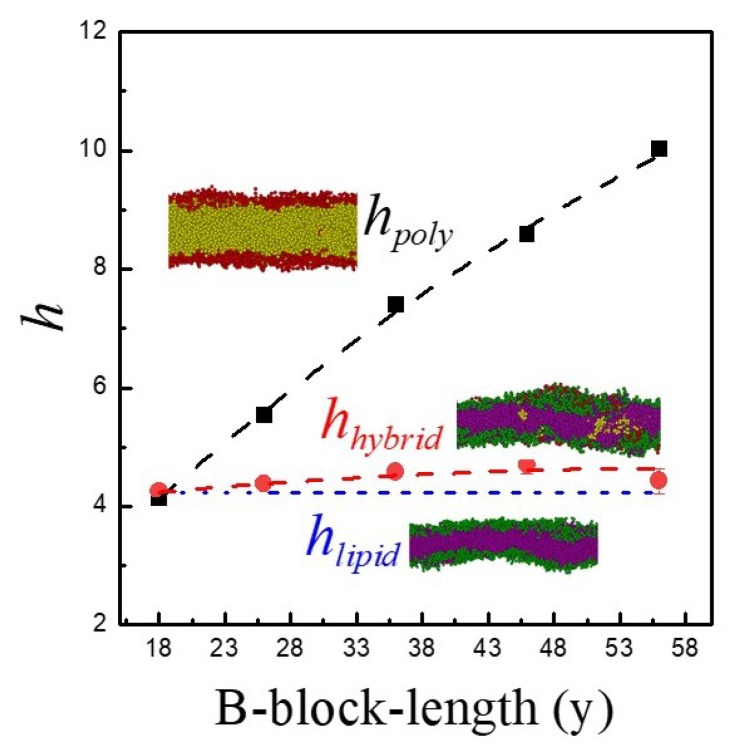
Thickness of hydrophobic layers of pure polymer membrane (*h_poly_*), pure lipid bilayer (*h_lipid_*), and hybrid membrane (*h_hybrid_*). The hybrid membrane is formed by lipids and A_3_B_y_A_3_ with $\varphi_{p}=0.1$. Green and purple beads represent lipid head and tail, respectively. Red and yellow beads denote hydrophilic and hydrophobic parts of the copolymer, respectively.

Conformation change for A_3_B_36_A_3_ in the lipid/triblock hybrid membrane is allowed to take place and an equilibrium condition with $f_{L}^{*}\approx 0.51$ is eventually reached. Since triblock copolymers with different hydrophobic B-block lengths (y) have distinct degrees of conformational entropy, it is reasonable to expect that the equilibrium fraction of loop-shape copolymers ($f_{L}^{*}$) within the hybrid membrane varies with y accordingly. To demonstrate the influence of the hydrophobic block length, we first consider a membrane formed by pure triblock copolymers $\varphi_{p}=1$. As illustrated in Figure 8, $f_{L}^{*}$ increases as the B-block length (y) grows from 18 to 56. It was reported that copolymers with loop-shape occupy more volume than those with bridge-shape [30]. At a given polymer length, larger occupied volume generally leads to higher entropy. Thus, as y increases, it is thermodynamically more preferable for the copolymer to take the loop-conformation in the pure polymeric membrane. However, for the hybrid membrane of lipids and A_3_B_y_A_3_ with $\varphi_{p}=0.1$, $f_{L}^{*}$ declines slightly with increasing y, as also shown in Figure 8. This opposite trend may be caused by the effect of size mismatch between lipids and copolymers. Figure 9 demonstrates the thickness of the hydrophobic layer (h) determined from the mean distance between the two interfaces associated with the hydrophobic and hydrophilic layers. Here $h_{poly}$, $h_{lipid}$ and $h_{hybrid}$ denote thickness of hydrophobic layers of pure polymer membrane ($\varphi_{p}=1$), pure lipid bilayer ($\varphi_{p}=0$), and hybrid membrane ($\varphi_{p}=0.1$), respectively. The thickness of the polymeric membrane ($h_{poly}$) is comparable to the thickness of the pure lipid bilayer ($h_{lipid}$) only for A_3_B_18_A_3_. As y rises, $h_{poly}$ becomes greater than $h_{lipid}$. A pure lipid bilayer has a rather compact structure. In a hybrid membrane with low $\varphi_{p}$, copolymers have to adapt their conformations to alleviate the size mismatch effect. Since bridge-shape copolymers take up less space than loop-shape ones, they are more comparable in size with lipids. As a result, more bridge-shape copolymers tend to develop as y increases, leading to a decrease in $f_{L}^{*}$. Note that in principle, it is possible to have a direct comparison between our coarse-grained DPD simulations and real experimental systems. However, the characteristic properties of the polymer/lipid models such as the interaction parameters between different species ($a_{ij}$), the cutoff distance (r_c_), and DPD bead mass (m) must be meticulously chosen, unlike microscopic MD simulations. In general, mesoscale DPD simulations can capture the essential features of macroscopic realistic cases. Our work clearly demonstrates that for a fully compatible systems of lipids and triblock copolymers, symmetric membranes always result for triblock polymers with short hydrophilic lengths. However, asymmetric membranes can be easily constructed for triblock copolymers with long hydrophilic lengths. This outcome is useful for the precise control of the development of new membranes with symmetric or asymmetric characteristics.

## 4. Conclusions

In this work, homogeneous hybrid membranes formed by co-assembly of triblock copolymers (A_x_B_y_A_x_) with lipid bilayers (H_3_(T_6_)_2_) were studied by dissipative particle dynamics. The polymer conformations can be generally categorized as bridge- and loop-shapes in the membranes. The hybrid membrane is called symmetric as the fractions of the loop-shape copolymers in the upper and lower leaflets are the same ($f_{L}^{up}=f_{L}^{lo}$), and it is termed asymmetric as $f_{L}^{up}\neq f_{L}^{lo}$. It was found that the long-time fraction of loop-conformation ($f_{L}$) of copolymers in the membrane depends significantly on the hydrophilic block length (x). As x is large, the structural characteristics of hybrid membranes persist throughout the simulation process and are essentially time-invariant. Both flip-flops and loop-bridge shape transformations are inhibited by the energy barriers associated with the strong repulsive interactions between the hydrophobic lipid tails and the long hydrophilic A-blocks ($∼x\cdot a_{AT}$). Consequently, the hybrid membranes involving copolymers with long hydrophilic blocks (e.g., A_10_B_56_A_10_) tend to be kinetically trapped in their original configurations and conformations. This outcome signifies that by co-assembling triblock copolymers with long hydrophilic A-blocks into lipid bilayers, asymmetric membranes with distinct initial values of $f_{L}^{up}$ and $f_{L}^{lo}$ can be designed and exist stably for a long period of time.

As x is small, the energy barrier of a short hydrophilic A-block moving across the hydrophobic layer is significantly smaller that of a long A-block. Both flip-flops between leaflets and conformation interchanges between loops and bridges take place frequently during the time evolution. As a result, the hybrid membrane of triblock copolymers with short hydrophilic A-blocks tends to evolve toward a true equilibrium state with symmetric configuration and specific $f_{L}^{*}$, irrespective of the initial conformation distribution. It was also found that $f_{L}^{*}$ depends on the hydrophobic block length (y) because the size mismatch between triblock copolymers and lipids grows as y increases. In a hybrid membrane with low $\varphi_{p}$, more bridge-shape copolymers tend to develop as y increases, leading to a decrease in $f_{L}^{*}$. This outcome can be attributed to the fact that bridge-shape copolymers take up less space than loop-shape ones and they are more comparable in size with lipids.
